# The Classification and Language Description of Patients with Primary Progressive Aphasia Using the Mini Linguistic State Examination Test

**DOI:** 10.3390/geriatrics10010002

**Published:** 2024-12-26

**Authors:** Elena Herrera, Claudia Acevedo, María González-Nosti

**Affiliations:** Department of Psychology, University of Oviedo, 33003 Oviedo, Spaingonzaleznmaria@uniovi.es (M.G.-N.)

**Keywords:** primary progressive aphasia, PPA semantic variant, PPA logopenic variant, PPA non-fluent variant, mini linguistic state examination Spanish version

## Abstract

**Introduction.** Primary progressive aphasia (PPA) is a clinical syndrome characterized by a progressive deterioration in language and speech. It is classified into three variants based on symptom patterns: logopenic, semantic, and non-fluent. Due to the lack of fully reliable and valid screening tests for diagnosing PPA and its variants, a Spanish version of the Mini Linguistic State Examination (MLSE) has recently been introduced. **Materials and methods.** This study aimed to describe the language impairments in a small sample of six patients with PPA and classify them into the three variants using the decision tree and syndrome guide proposed by the MLSE authors. **Results.** The findings demonstrate the test’s utility in classifying some PPA variants through a qualitative analysis of patient performance and error types. The study revealed a 50% accuracy rate for the decision tree and an 83.33% accuracy rate when using the syndrome guide. **Discussion.** This discrepancy arises because the decision tree often classified cases as logopenic variant PPA (lvPPA) when working memory was significantly impaired. Specifically, it tended to misclassify patients with semantic, motor, or speech impairments as having lvPPA due to its reliance on the sentence repetition task for assessing working memory.

## 1. Introduction

Primary progressive aphasia (PPA) is a clinical syndrome with heterogeneous neuropathological causes, characterized by a relatively focal degeneration of the brain systems involved in language, resulting in a progressive decline in language and speech. Currently, three variants of PPA are identified [1]: non-fluent or agrammatic PPA (nfvPPA), semantic PPA (svPPA), both associated with frontotemporal lobar degeneration (FTLD), and logopenic PPA (lvPPA), mostly associated with Alzheimer’s Disease (AD) [2].

Anatomically, nfvPPA is usually linked with atrophy of the left perisylvian cortex, centered on the inferior frontal gyrus and anterior insula [3], and local network changes in frontal brain regions such as the pars opercularis and the middle frontal cortex [4]. nfvPPA is an early-onset syndrome, with a mean age of onset around 60 years [5]. The two main features of this variant are the presence of agrammatism in language production (characterized by short sentences and omissions of grammatical morphemes) and/or comprehension, and apraxia of speech (strained and choppy speech with speech sound distortions and errors). One of these features must be present to diagnose this type of PPA [1]. Patients frequently exhibit distortions, substitutions, omissions, and additions of phonemes, their prosody and speech rate may be impaired, and apraxia of speech may be present [6]. Additionally, sentence comprehension, especially of syntactically complex sentences, may be affected [7]. However, both single-word comprehension and object knowledge are usually preserved. Two of these three features must also be present for a diagnosis. Many patients with nfvPPA eventually develop Parkinsonism or other motor-based disorders [3], although the mild impairment of fine hand movements or mild apraxia is not exclusionary [1].

In the case of svPPA, pathology is most commonly associated with atrophy of the antero-mesial temporal lobe (initially more predominant on the left side) [3] and reduced connectivity and impaired information processing in temporal and limbic brain areas [4]. The two main features of this variant, both essential for diagnosis, are anomia and word comprehension difficulties, predominantly in single words. Word frequency (defined as the number of occurrences per million in an oral or written corpus) is the key variable in the performance of these patients [8], as lexical retrieval is facilitated by the most available words in the lexicon [9]. Object recognition is also often impaired and is affected by familiarity, with objects that the patient frequently encounters being the most easily recognizable [10]. Regarding written language, surface dyslexia and dysgraphia are frequent, as patients use sublexical mechanisms to write or read, resulting in frequent regularization in reading and spelling errors in writing [11]. However, these features can only be readily identified in languages with irregular orthography, such as English. To detect surface dyslexia and dysgraphia in Spanish, it is often necessary to include irregular words in the evaluation, since Spanish orthography is generally regular [12]. Repetition and speech production, on the other hand, are preserved in these patients. Three of these four features must be present for a diagnosis [1].

Finally, lvPPA is typically associated with AD and has the greatest consistency between underlying pathology and symptomatology [13]. At the brain level, lvPPA is usually associated with atrophy of the left temporo-parietal regions [3]. This pattern is related to anatomical changes in partly overlapping functional networks anchored to two left temporo-parietal epicenters with an asymmetric pattern of atrophy characterized by the cortical involvement of left-hemisphere structures [14]. The most prominent features are impaired word retrieval in both spontaneous speech and naming, and the impaired repetition of phrases and sentences. Both symptoms are required for diagnosis [1]. Given the relationship between lvPPA and a deficit in phonological short-term memory, the repetition and comprehension of phrases and sentences are more impaired than that of single words [15]. Additional features, such as phonological errors in naming and frequent pauses during spontaneous speech to perform lexical retrieval, may be present, but there is no evident agrammatism. The motor aspects of speech are preserved as well as the comprehension of single words and the semantic knowledge of objects. At least three of these features must be present to make a diagnosis [1].

To these described variants, Mesulam et al. [16] add mixed PPA, often associated with AD, referring to patients who both have comprehension deficits and make grammatical errors. Regarding the evolution of PPA, it is common for some patients who initially meet the criteria for one variant to eventually develop alterations in other language domains not characteristic of the previously diagnosed variant; Louwersheimer et al. [17] refer to these cases as extended PPA or unclassifiable patients [18].

Given the variability in the onset and subsequent evolution of the disease, it is of utmost importance to make an early diagnosis when symptoms begin to manifest and only some linguistic domains are affected. In this way, specific pharmacological [19,20,21,22] (only for managing the behavioral or mood aspects of PPA) and non-pharmacological (e.g., speech therapy) treatments [23,24] can be initiated to slow down the cognitive and language deficits associated with the disease. However, there are currently not many assessment tools available that allow an accurate diagnosis of the different variants and follow-up with patients. Some widely accepted tools are the Sydney Language Battery [25] and the Screening for Aphasia in Neurodegeneration [26], but none have a version adapted to Spanish.

Recently, the Mini Linguistic State Examination (MLSE), which assesses the main linguistic domains affected by PPA according to the diagnostic criteria [1], has been developed [27]. It consists of eleven subtests that assess the following language skills: naming, word and sentence repetition, word and sentence comprehension, semantic association, reading, writing, and a connected speech task. In each subtest, the different types of errors are collected (speech-motor, phonology, semantics, syntax, and auditory–verbal working memory), which are taken into account later for the calculation of the score in each domain, from which the subject’s variant is determined and the overall score, which provides information about the severity of the disorder.

The MLSE is the only PPA-specific test available with a Spanish version [28]. The tasks and items are consistent with the original version, except for some modifications reflecting the psycholinguistic features of the stimuli used. For instance, the task of reading regular and irregular words was replaced in the Spanish version by reading words without a tilde, a grammatical mark indicating the stressed syllable, as all Spanish words are regular.

The original article introducing the MLSE presents a decision tree based on scores across various language domains, designed to aid in the diagnostic process. In addition to this, the test includes a syndrome guide that outlines the characteristics of each PPA variant. However, despite the availability of these diagnostic tools, no subsequent studies in Spanish have employed this instrument or decision tree to classify patients and describe the language impairments associated with each PPA variant.

Thus, the objective of this study was to describe a small sample of Spanish patients with PPA using the recently published MLSE. Furthermore, the decision tree proposed by Matias-Guiu et al. [28] and the syndrome guide within the MLSE were employed to evaluate the concordance between these tools in classifying the three PPA variants.

## 2. Materials and Methods

### 2.1. Participants

A total of 6 patients (1 male and 5 females), ranging in age from 59 to 83 years old, with a clinical diagnosis of PPA [1] in one of its variants (lvPPA, svPPA, and nfvPPA) took part in this study. All participants were diagnosed by a specialist neurologist, and the diagnosis was confirmed by neuroimaging techniques.

The characteristics of the onset of symptoms and other relevant information were gathered from the patients’ clinical history and during the interview conducted for the neuropsychological evaluation. The relevant characteristics of the subjects, collected during the interview, the application of the neuropsychological protocol, and their clinical histories are described below and summarized in Table 1.

Subject 1 was a 78-year-old woman diagnosed with svPPA. She left school at the age of 14 and was engaged in cattle raising throughout her working life. While the precise onset of symptoms is unclear, her family reported noticing difficulties in comprehending conversations about 5 years before the evaluation. The clinical diagnosis was confirmed two years prior to the assessment.

Subject 2, a 69-year-old man with significant language difficulties related to word retrieval and AD pathology, achieved a graduate-level degree in mathematics. Symptoms manifested approximately 4 years before the evaluation, coinciding with his retirement. Initial symptoms included difficulties in naming common objects and occasional challenges in pronunciation, as reported by his family.

Subject 3, an 83-year-old woman diagnosed with nfvPPA, received a primary school education and worked as a sales assistant. Symptoms began approximately 5 years ago, characterized by difficulties in articulating words, frequent pauses, and fragmented speech, as described by her family. The clinical diagnosis was established 2 years before the evaluation.

Subject 4, a 73-year-old woman diagnosed with AD and lvPPA, received a primary school education and worked in a plug factory. Symptoms started around 4 years before the evaluation, coinciding with the onset of some depressive symptoms. One of the initial signs observed was difficulty in oral expression, accompanied by frequent instances of the tip-of-the-tongue phenomenon.

Subject 5 was a 73-year-old woman diagnosed with nfvPPA and frontotemporal atrophy-type cognitive impairment. She left school at the age of 14 and had worked for more than 50 years in a tailor’s shop. At the time of the assessment, symptoms had been present for approximately four years.

Finally, Subject 6 was a 59-year-old woman diagnosed 2 years before the evaluation with PPA in the context of frontotemporal dementia. She completed 12 years of education but was not subsequently engaged in paid employment. While she did not have a specific diagnosis of any of the three variants, svPPA was suspected due to predominant anomic difficulties.

### 2.2. Instruments

The patients were evaluated using the Spanish version of the MLSE [28], which consists of eleven subtasks designed to assess the primary linguistic domains affected by PPA: a motor speech component, semantic knowledge, phonology, syntax, and working memory. These subtasks include picture naming, syllable and word repetition, repetition and pointing out, pseudoword repetition, semantic association, sentence comprehension, sentence comprehension with pictures, word and pseudoword reading, sentence repetition, and spoken picture description.

From the participants’ responses, two scores were derived: one was used to identify the nature of the linguistic impairment and determine the specific variant of PPA based on the number of errors in each linguistic domain, thereby providing a profile of scores across domains. The other score, rated out of 100, assesses the overall degree of language impairment and is particularly useful for screening and follow-up purposes. A higher overall score indicates more severe language impairment.

**Figure 1:**
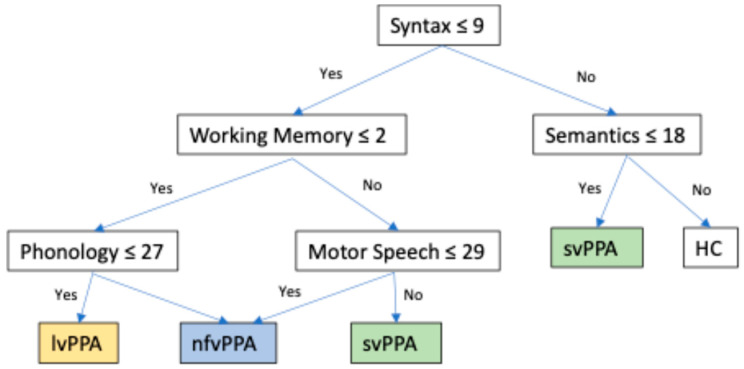
Decision tree developed by Matias-Guiu et al. (2021) [28]. svPPA—semantic variant PPA; nfvPPA—non-fluent PPA; lvPPA—logopenic variant PPA. HC = healthy control.

The decision tree developed by Matias-Guiu et al. [28], attached below in Figure 1, serves as a valuable tool to aid in the diagnosis of PPA variants, guiding clinicians based on the scores obtained in each language domain. Additionally, the MLSE provides a syndrome guide based on its tasks, which further aids in the classification of patients (see Table 2).

### 2.3. Procedure

Patient recruitment was conducted through the Neurology Service of the Hospital de Cabueñes (Asturias), where five of the participants were evaluated; the sixth participant was assessed at the rehabilitation clinic he regularly attended. Each evaluation session lasted approximately 30 min and was conducted in a room free from visual and auditory distractions by a specialist in neuropsychology. The session began with the collection of general data (neurological diagnosis, duration of symptoms, age, etc.), followed by the administration of the MLSE test.

All participants or their relatives were informed about the project’s objectives and signed an informed consent form. They also agreed to the recording of the evaluation session to enable a detailed analysis of the patients’ responses.

To ensure the accuracy of the test scores and the classification of errors, all evaluations were overseen by the principal investigator of this project.

## 3. Results

After the patients were evaluated using the MLSE test, a descriptive analysis of the results obtained in each subtest was performed. These evaluation results are summarized in Table 3, while examples of language production and the corresponding types of errors are presented in Table 4.

### 3.1. Subject A: svPPA

This patient was initially diagnosed with svPPA and obtained a global score of 54/100. Performance in each domain was particularly low in semantics (1/20), syntax (0/20), and working memory (0/20), although she also scored poorly in phonology (24/30). Additionally, the patient’s comprehension was highly impaired, with significant difficulties in understanding task instructions. In the word reading task, she made regularization errors in three out of six words. In the picture naming and semantic association tasks, she failed due to a lack of understanding of the concepts, even when the examiner provided the correct answer. She was unable to describe the use or any characteristics of the word. Similar difficulties were observed in other tasks, such as sentence comprehension and sentence repetition, in which she did not fully grasp the nature of the task and attempted to give an answer rather than correctly identify the image or repeat the sentence.

Although the decision tree led to an lvPPA diagnosis, after the results were analyzed, the diagnosis proposed is svPPA. This conclusion is due to the significant deficits in semantics, syntax, and working memory, including impairment in comprehension.

### 3.2. Subject B: From PPA + AD to lvPPA

Patient B did not have a specific clinical classification for PPA but was also diagnosed with AD. However, the clinical history suggests that he initially presented with a marked language disorder including impairment in naming and phonological errors indicative of lvPPA. After the MLSE was administered, the worst scores were in working memory (2/10), phonology (18/30), and semantics (14/20), and he obtained a global score of 73/100. The errors observed during this patient’s evaluation were primarily phonological, occurring in tasks such as picture naming, the repetition of syllables, words, and pseudowords, and pseudoword reading. He also exhibited semantic errors in picture naming and made 2/6 regularization errors in the word reading task. Additionally, he faced significant difficulties in the sentence repetition task.

The proposed diagnosis, after the results were analyzed, is lvPPA, consistent with the decision tree and his clinical history. This diagnosis aligns with the fact that lvPPA is predictive of AD in most cases.

### 3.3. Subject C: From nfvPPA to svPPA

This patient was diagnosed with nfvPPA and showed the worst performance in the domains of working memory and syntax, scoring 0/10 in each, and in semantics (7/20). Phonology (24/30) was also impaired, though to a lesser extent. The overall score was 61/100. Additionally, there were failures in the semantic domain and numerous problems in understanding task instructions, often requiring multiple explanations. Spontaneous speech was notably deficient, characterized by constant pauses, stuttering, and hesitations. The most frequent errors were semantic, occurring in tasks such as picture naming, word–picture matching, and semantic association. In the sentence comprehension task, the patient struggled due to difficulties in understanding the task itself.

Although the decision tree suggested an lvPPA diagnosis, the proposed diagnosis is svPPA due to impairments in linguistic domains not typical of lvPPA, such as comprehension, along with the frequent semantic errors and pauses in spontaneous speech. The initial clinical diagnosis was nfvPPA, but it appears that the syntactic errors may have been due to comprehension difficulties.

### 3.4. Subject D: lvPPA

In Subject D, diagnosed with lvPPA, the most impaired domain was working memory (0/10). There were also difficulties in syntax (5/10), semantics (13/20), and phonology (23/30). The global score was 71/100. Spontaneous speech was quite fluent but marked by frequent pauses for word retrieval, as well as numerous circumlocutions and generalizations. This patient exhibited phonological errors in the picture-naming task, as well as in repetition and reading of pseudowords. Semantic errors were primarily observed in the picture naming task and in the picture description task. The patient also made 2/6 regularization errors in the word reading task. Syntactic errors were present in the sentence comprehension task.

After the results were analyzed, the proposed diagnosis remains lvPPA, consistent with the decision tree and the initial clinical diagnosis.

### 3.5. Subject E: nfvPPA

This patient was diagnosed with nfvPPA and had a global score of 51/100. The speech (motor) domain was particularly compromised (12/30), with distorted speech during the interview, marked by stereotypies and perseverations. For example, during picture naming tasks, she repeated the same response multiple times. She was unable to formulate phrases or sentences and often spoke in whispers, further complicating comprehension. These errors were present across all test tasks that required an oral response.

Other linguistic domains were also affected, with scores of 25/30 in phonology, 13/20 in semantics, 0/10 in working memory, and 1/10 in syntax. Based on these results, the proposed diagnosis is nfvPPA according to the initial diagnosis, although the decision tree pointed toward the lvPPA variant.

### 3.6. Subject F: From PPA + FTD to svPPA

Finally, Subject F was diagnosed with unclassified PPA in the context of frontotemporal dementia. The global score was 69/100, and the most impaired domains were semantics (8/20), followed by working memory (0/10) and syntax (3/10). During the interview, her language was quite fluent, as she could formulate phrases and sentences, but was marked by difficulty in finding words, often substituting them with more generic words and/or circumlocutions. The patient also exhibited numerous verbal stereotypies such as “piu, piu” or “curu, curu” and frequent prosodic errors in the reading task, suggesting a loss of visual representations in semantic memory. Semantic errors occurred not only in tasks requiring oral word production, such as picture naming or picture description, but also in tasks involving semantic association.

Although the decision tree suggested nfvPPA, after the MLSE results and clinical observations were analyzed, the inclination is toward an svPPA diagnosis.

## 4. Discussion

The development of the MLSE [27] has been a significant contribution to the assessment of PPA, a clinical syndrome characterized by a gradual decline in language and speech abilities. Notably, the MLSE is the only specific test designed to evaluate the three PPA variants (logopenic, non-fluent, and semantic) with a recently developed Spanish version [28]. The primary aim of this study was to describe the performance of a small sample of Spanish patients with PPA using both the decision tree and the syndrome guide included in the MLSE. Additionally, this study aimed to determine whether there was concordance between these two tools in classifying the patients.

Overall, the results indicate that the test is effective in classifying the three variants of PPA. In this study, the decision tree showed an accuracy rate of 50%, compared to the 72% accuracy reported by the MLSE authors [28]. However, when the syndrome guide was used, 83.3% of the patients were correctly classified.

The analysis of the classifications obtained using both the decision tree and the syndrome guide included in the MLSE test provides valuable insights. It was found that the diagnosis was aligned across the initial clinical diagnosis, the decision tree, and the syndrome guide in only one case of lvPPA. Interestingly, there was concordance between the patient’s initial diagnosis and the syndrome guide in five out of the six cases. In contrast, when the decision tree was applied, this alignment was observed in only three of the six cases. A notable discrepancy occurred in one case (Subject C), in which the initial diagnosis was nfvPPA, but the decision tree classified the patient as having lvPPA, while the syndrome guide indicated a diagnosis of svPPA. These results suggest that the decision tree may be less effective for accurately classifying PPA patients compared to the syndrome guide, which appears to be the more reliable tool.

This issue likely arises because the decision tree tends to classify cases as lvPPA when significant impairments in working memory are detected, as lvPPA is characterized by deficits in this cognitive domain [15]. Moreover, this could be attributed to the higher prevalence of this subtype compared to others [29] or to the fact that working memory is evaluated exclusively through the sentence repetition task, which requires the repetition of increasingly complex sentences. Observations of the patients’ performance suggest that many individuals with semantic, motor, or speech impairments struggle to accurately repeat sentences due to these difficulties. For instance, one case, initially diagnosed with svPPA and correctly classified by the syndrome guide, was categorized as lvPPA by the decision tree. Given that svPPA is associated with severe deficits in word comprehension [1], it is likely that difficulties in understanding specific words within the sentences hindered the patient’s ability to repeat them correctly, leading to misclassification as a working memory disorder. Similarly, both patients initially diagnosed with nfvPPA were reclassified as having lvPPA by the decision tree, while the syndrome guide classified one as having svPPA and retained the nfvPPA diagnosis for the other. Although diagnostic changes can occur over time with follow-up assessments, studies suggest that in the cases of nfvPPA and svPPA, the diagnosis tends to remain stable over time [30]. In this instance, one of the patients initially diagnosed with nfvPPA exhibited language characteristics more aligned with svPPA. This discrepancy could be due to a misinterpretation of the deficits or because difficulties in word comprehension and production led to hesitations or articulatory errors that may have interfered with the interpretation of the patient’s symptoms.

Although the sentence repetition task is appropriate for assessing working memory, it may be beneficial to include an additional brief task to evaluate this domain with a reduced verbal load, enabling a clearer distinction between motor deficits and pure working memory impairments. Previous studies have demonstrated that working memory tends to decline as nfvPPA progresses, with this impairment becoming more evident over time. In contrast, in lvPPA, working memory deficits are present from the early stages, as evidenced by other evaluation tasks [31]

The rapid evolution of the disease, progressing from isolated language alterations typical of PPA to global cognitive impairment with multiple neuropsychiatric symptoms [32], underscores the importance of early diagnosis when language deficits are isolated and the variant can be accurately identified. Early diagnosis enhances the likelihood of providing appropriate clinical interventions, controlling symptoms, reducing costs, and delaying institutionalization [33].

The establishment of a reliable diagnosis is challenging due to multiple factors, including the variable progression of the disease and the presence of different variants of PPA. In some patients, language may remain the sole impaired domain for up to ten years. An individual protective factor influencing disease progression is the number of years of education; more years of education is associated with slower disease progression, potentially explained by the cognitive reserve hypothesis [34] or early symptom detection.

Interestingly, all patients in the sample had a low level of education, except for one subject, a mathematician with a university education who began exhibiting symptoms three years ago, coinciding with his retirement. Further in-depth studies on the importance of measuring patients’ premorbid conditions and their association with disease progression may provide additional insights.

The progression of PPA varies by variant. For example, progression is faster in lvPPA than in svPPA or nfvPPA. However, patients with svPPA experience a longer duration between symptom onset and diagnosis [35]. This delay can be attributed to the masking of semantic knowledge loss by circumlocutions or synonyms, preventing family members from recognizing the disease. In contrast, nfvPPA often triggers alarms due to slurred speech and lvPPA due to poor word retrieval in spontaneous speech [32].

An awareness of language proficiency changes, typically occurring in individuals in their 50s, is crucial for facilitating early diagnosis. Alarm symptoms include mild and persistent pauses in word search (particularly for low-frequency words), anomia, syntactic anomalies, poor spelling, and comprehension failures [16].

One limitation of this study is its small sample size of just six patients, as it is essentially a case study, which restricts the generalizability of the results. Nonetheless, this preliminary investigation into the test and its diagnostic predictions, through detailed patient studies, has highlighted a major weakness of the test and proposed a potential solution to address this bias. Specifically, it suggests evaluating working memory with a different test that does not have a high linguistic load, which may hinder patients from performing accurately due to their difficulties with language comprehension or production.

## 5. Conclusions

In conclusion, this study provides valuable insights into the classification of PPA variants using the MLSE, demonstrating its effectiveness as a quick, easy-to-administer diagnostic tool. However, the findings also underscore the need for the further refinement of diagnostic methods to enhance accuracy. While the MLSE proved effective in classifying PPA variants, with the syndrome guide more reliably aligning with initial diagnoses, the decision tree exhibited some limitations. Specifically, it tended to misclassify patients with semantic, motor, or speech impairments as having lvPPA due to its reliance on the sentence repetition task for assessing working memory.

One key takeaway from this study is the need to incorporate alternative tasks for evaluating working memory, especially those with a reduced verbal load. This would help avoid misclassification related to language production or comprehension difficulties and allow for a clearer distinction between motor or semantic deficits and true working memory impairments.

Despite the small sample size, the present research highlights the MLSE’s potential as a valuable tool for diagnosing PPA, particularly when complemented by thorough clinical assessments. Future studies should focus on addressing the identified limitations and examining the progression of PPA variants in larger cohorts. Early and accurate diagnosis remains essential for providing timely interventions, managing symptoms, and ultimately improving patients’ quality of life.

## Figures and Tables

**Table 1:** Demographic and clinical data of the participants.

Subject	Gender	Age	Time from Diagnosis	Years of School
Subject A	Female	78	2 years	8
Subject B	Male	69	3 years	17
Subject C	Female	83	2 years	6
Subject D	Female	73	3 years	6
Subject E	Female	73	3 years	8
Subject F	Female	59	2 years	12

**Table 2:** Syndrome guide included in the MLSE.

PPA Classification	Mot.	Phon.	Sem.	Syn.	WM
svPPA	Normal	Normal	Impaired	Normal or mild impairment	Normal
nfvPPA	Impaired	(and/or) Impaired	Normal	(and/or) Impaired	Normal
lvPPA	Normal	Impaired	Mild impairment	Mild impairment	Impaired

PPA—primary progressive aphasia; svPPA—semantic variant PPA; nfvPPA—non-fluent PPA; lvPPA—logopenic variant PPA. Mot.—motor component of speech; Phon.—phonology; Sem.—semantic knowledge; Syn.—syntax; WM—working memory.

**Table 3:** Summary of scores in each test domain for each participant including initial diagnosis, diagnosis based on decision tree, and diagnosis by MLSE description.

	Scores by MLSE Domain (% of Errors)									
Subj.	Mot. (30)	Phon. (30)	Sem. (20)	Syn. (10)	WM (10)	NR	Total Score	Initial Diagnosis	Diagnosis by Dec. Tree	Diagnosis by MLSE Description
Subj. A	29 (3.3%)	24 (20%)	1 (95%)	0 (100%)	0 (100%)	0 (0%)	54	svPPA	lvPPA	svPPA
Subj. B	29 (3.3%)	18 (40%)	14 (30%)	9 (10%)	2 (80%)	0 (0%)	73	PPA + AD	lvPPA	lvPPA
Subj. C	30 (0%)	24 (20%)	7 (65%)	0 (100%)	0 (100%)	0 (0%)	61	nfvPPA	lvPPA	svPPA
Subj. D	30 (0%)	23 (23.3%)	13 (35%)	5 (59%)	0 (100%)	0 (0%)	71	lvPPA	lvPPA	lvPPA
Subj. E	12 (60%)	25 (16.6%)	13 (35%)	1 (90%)	0 (100%)	0 (0%)	51	nfvPPA	lvPPA	nfvPPA
Subj. F	30 (0%)	28 (6.6%)	8 (60%)	3 (70%)	0 (100%)	0 (0%)	69	PPA + FTD	svPPA	svPPA

Subj.—subject; Mot.—motor component of speech; Phon.—phonology; Sem.—semantic knowledge; Syn.—syntax; WM—working memory; NR—non-response; dec. tree—decision tree; PPA—primary progressive aphasia; nfvPPA—non-fluent PPA; svPPA—semantic PPA; lvPPA—logopenic PPA. Maximum scores for each domain are in parentheses.

**Table 4:** Examples of patients’ oral production samples along with the type of error.

Language Samples				
Subj.	Naming (CR)/ann.	Repetition (CR)/ann.	Reading Words/ann.	Reading Pseu (CR)/ann.
A	NR	CR	visPEra/reg	Perece (perace)/phon.
B	Peña (piña)/phon.	Fulberupon (fubelpuron)/phon	CR	Ejeno (ejono)/phon.
C	cabaña (iglú)/sem	CR	feREtro/reg	CR
D	Endubo (embudo)/phon.	Fubalpural (fubelpuron)/phon	CR	Gujano (gujamo)/phon.
E	Dist.	Dist.	Dist.	Jugamo (gujamo)phon.
F	NR	CR	soLIdo/reg	CR

Note: ann—annotation (type of error); CR—correct response; NR—non-response; Reading pseu—reading pseudowords; reg—regularization error (the emphasis when reading has been indicated with uppercase letters); phon.—phonologic error; dist—distortions.

## Data Availability

Data will be made available upon reasonable request to herreraelena@uniovi.es.

## References

[1] Gorno-Tempini M.L., Hillis A.E., Weintraub S., Kertesz A., Mendez M., Cappa S.F., Ogar J.M., Rohrer J.D., Black S., Boeve B.F. (2011). Classification of primary progressive aphasia and its variants. Neurology.

[2] Mesulam M., Wicklund A., Johnson N., Rogalski E., Léger G.C., Rademaker A., Weintraub S., Bigio E.H. (2008). Alzheimer and Frontotemporal Pathology in Subsets of Primary Progressive Aphasia. Ann. Neurol..

[3] Marshall C.R., Hardy C.J.D., Volkmer A., Russell L.L., Bond R.L., Fletcher P.D., Clark C.N., Mummery C.J., Schott J.M., Rossor M.N. (2018). Primary progressive aphasia: A clinical approach. J. Neurol..

[4] Nigro S., Tafuri B., Urso D., De Blasi R., Cedola A., Gigli G., Logroscino G., Initiative F.T.F.L.D.N. (2021). Altered structural brain networks in linguistic variants of frontotemporal dementia. Brain Imaging Behav..

[5] Johnson J.K., Diehl J., Mendez M.F., Neuhaus J., Shapira J.S., Forman M., Chute D.J., Roberson E.D., Pace-Savitsky C., Neumann M. (2005). Frontotemporal Lobar Degeneration: Demographic Characteristics of 353 Patients. Arch. Neurol..

[6] Matías-Guiu J., García-Ramos R. (2013). Afasia progresiva primaria: Del síndrome a la enfermedad. Neurol..

[7] Thompson C.K., Mack J.E. (2014). Grammatical impairments in PPA. Aphasiology.

[8] Caine D., Breen N., Patterson K. (2009). Emergence and progression of ‘non-semantic’ deficits in semantic dementia. Cortex.

[9] Stockbridge M.D., Venezia J.H., Vitti E., Tippett D.C., Hillis A.E. (2022). Verb Frequency and Density Drive Naming Performance in Primary Progressive Aphasia. Aphasiology.

[10] Hodges J.R., Patterson K., Oxbury S., Funnell E. (1992). Semantic dementia: Progressive fluent aphasia with temporal lobe atrophy. Brain.

[11] Brambati S., Ogar J., Neuhaus J., Miller B., Gorno-Tempini M. (2009). Reading disorders in primary progressive aphasia: A behavioral and neuroimaging study. Neuropsychologia.

[12] Matías-Guiu J.A., Cuetos F., Cabrera-Martín M.N., Valles-Salgado M., Moreno-Ramos T., Carreras J.L., Matías-Guiu J. (2017). Reading difficulties in primary progressive aphasia in a regular language-speaking cohort of patients. Neuropsychologia.

[13] Spinelli E.G., Mandelli M.L., Miller Z.A., Santos-Santos M.A., Wilson S.M., Agosta F., Grinberg L.T., Huang E.J., Trojanowski J.Q., Meyer M. (2017). Typical and atypical pathology in primary progressive aphasia variants. Ann. Neurol..

[14] Mandelli M.L., Lorca-Puls D.L., Lukic S., Montembeault M., Gajardo-Vidal A., Licata A., Scheffler A., Battistella G., Grasso S.M., Bogley R. (2023). Network anatomy in logopenic variant of primary progressive aphasia. Hum. Brain Mapp..

[15] Macoir J., Macoir J., Laforce R., Laforce R., Lavoie M., Lavoie M. (2023). The impact of phonological short-term memory impairment on verbal repetition in the logopenic variant of primary progressive aphasia. Aging Neuropsychol. Cogn..

[16] Mesulam M.-M., Rogalski E.J., Wieneke C., Hurley R.S., Geula C., Bigio E.H., Thompson C.K., Weintraub S. (2014). Primary progressive aphasia and the evolving neurology of the language network. Nat. Rev. Neurol..

[17] Louwersheimer E., Keulen M.A., Steenwijk M.D., Wattjes M.P., Jiskoot L.C., Vrenken H., Teunissen C.E., van Berckel B.N., van der Flier W.M., Scheltens P. (2016). Heterogeneous Language Profiles in Patients with Primary Progressive Aphasia due to Alzheimer’s Disease. J. Alzheimer’s Dis..

[18] Utianski R.L., Botha H., Martin P.R., Schwarz C.G., Duffy J.R., Clark H.M., Machulda M.M., Butts A.M., Lowe V.J., Jack C.R. (2019). Clinical and neuroimaging characteristics of clinically unclassifiable primary progressive aphasia. Brain Lang..

[19] Johnson N.A., Rademaker A., Weintraub S., Gitelman D., Wienecke C.B., Mesulam M. (2010). Pilot trial of memantine in primary progressive aphasia. Alzheimer Dis. Assoc. Disord..

[20] Boxer A.L., Lipton A.M., Womack K., Merrilees J.R., Neuhaus J., Pavlic D.B., Gandhi A.B., Red D.B., Martin-Cook K.B., Svetlik D.R. (2009). An open-label study of memantine treatment in 3 subtypes of frontotemporal lobar degeneration. Alzheimer Dis. Assoc. Disord..

[21] Kertesz A., Morlog D., Light M., Blair M., Davidson W., Jesso S., Brashear R. (2008). Galantamine in Frontotemporal Dementia and Primary Progressive Aphasia. Dement. Geriatr. Cogn. Disord..

[22] Reed D.A., Johnson N.A., Thompson C., Weintraub S., Mesulam M.-M. (2004). A clinical trial of bromocriptine for treatment of primary progressive aphasia. Ann. Neurol..

[23] Roheger M., Riemann S., Brauer A., McGowan E., Grittner U., Flöel A., Meinzer M. (2024). Non-pharmacological interventions for improving language and communication in people with primary progressive aphasia. Cochrane Database Syst. Rev..

[24] Volkmer A., Rogalski E., Henry M., Taylor-Rubin C., Ruggero L., Khayum R., Kindell J., Gorno-Tempini M.L., Warren J.D., Rohrer J.D. (2019). Speech and language therapy approaches to managing primary progressive aphasia. Pract. Neurol..

[25] Savage S., Hsieh S., Leslie F., Foxe D., Piguet O., Hodges J.R. (2013). Distinguishing Subtypes in Primary Progressive Aphasia: Application of the Sydney Language Battery. Dement. Geriatr. Cogn. Disord..

[26] Catricalà E., Gobbi E., Battista P., Miozzo A., Polito C., Boschi V., Esposito V., Cuoco S., Barone P., Sorbi S. (2017). SAND: A screening for aphasia in neurodegeneration. development and normative data. Neurol. Sci..

[27] Patel N., Peterson K.A., Ingram R.U., Storey I., Cappa S.F., Catricala E., Halai A., Patterson K.E., Ralph M.A.L., Rowe J.B. (2021). A ‘Mini Linguistic State Examination’ to classify primary progressive aphasia. Brain Commun..

[28] Matias-Guiu J.A., Pytel V., Hernández-Lorenzo L., Patel N., Peterson K.A., Garrard P., Cuetos F. (2021). Spanish Version of the Mini-Linguistic State Examination for the Diagnosis of Primary Progressive Aphasia. J. Alzheimer’s Dis..

[29] Saracino D., Ferrieux S., Nogues-Lassiaille M., Houot M., Funkiewiez A., Sellami L., Deramecourt V., Pasquier F., Couratier P., Pariente J. (2021). Primary Progressive Aphasia Associated with GRN Mutations: New Insights Into the Nonamyloid Logopenic Variant. Neurology.

[30] Perry D.C., Datta S., Miller Z.A., Rankin K.P., Gorno-Tempini M.L., Kramer J.H., Rosen H.J., Seeley W.W., Miller B.L. (2019). Factors that predict diagnostic stability in neurodegenerative dementia. J. Neurol..

[31] Gorno-Tempini M.L., Dronkers N.F., Rankin K.P., Ogar M.J., Phengrasamy L., Rosen H.J., Johnson J.K., Weiner M.W., Miller B.L. (2004). Cognition and Anatomy in Three Variants of Primary Progressive Aphasia. Ann. Neurol..

[32] de la Sablonnière J., Tastevin M., Lavoie M., Laforce R. (2021). Longitudinal Changes in Cognition, Behaviours, and Functional Abilities in the Three Main Variants of Primary Progressive Aphasia: A Literature Review. Brain Sci..

[33] Mouton A., Plonka A., Fabre R., Tran T.M., Robert P., Macoir J., Manera V., Gros A. (2022). The course of primary progressive aphasia diagnosis: A cross-sectional study. Alzheimer’s Res. Ther..

[34] Perneczky R., Diehl-Schmid J., Pohl C., Drzezga A., Kurz A. (2007). Non-fluent progressive aphasia: Cerebral metabolic patterns and brain reserve. Brain Res..

[35] Matias-Guiu J.A., Cabrera-Martín M.N., Moreno-Ramos T., García-Ramos R., Porta-Etessam J., Carreras J.L., Matías-Guiu J. (2014). Clinical course of primary progressive aphasia: Clinical and FDG-PET patterns. J. Neurol..

